# Editorial: From microbial immunology to microbial therapeutics *via* targeted delivery technology

**DOI:** 10.3389/fimmu.2022.1033213

**Published:** 2022-09-23

**Authors:** Matteo Puccetti, César Viseras Iborra, Luigina Romani, Maurizio Ricci

**Affiliations:** ^1^ Department of Pharmaceutical Sciences, University of Perugia, Perugia, Italy; ^2^ Department of Pharmacy and Pharmaceutical Technology, University of Granada, Granada, Spain; ^3^ Department of Medicine and Surgery, University of Perugia, Perugia, Italy

**Keywords:** microbiota, metabolites, postbiotics, drug delivery, biopharmaceutics

An improved understanding of the multilevel interactions between microbes and the mammalian immune system has identified the crucial contribution of the microbiome to human health. Consequently, the intestinal microbiota has emerged as an attractive therapeutic target. The vast majority of microbiota-targeting therapies aim at engineering the intestinal ecosystem by means of probiotics or prebiotics (1). Recently, advances in high-throughput sequencing and metabolomics have led to the emergence of metabolic byproducts secreted by live bacteria that are master regulators of human health (2). Bioactive microbial metabolites – also referred to as postbiotics – have drawn attention because of their clear chemical structure, safety-dose parameters, long shelf life, and their ability to rescue gut health while preserving microbiota integrity. An emerging understanding of postbiotic metabolites is pointing to new frontiers in microbiome science together with an improved understanding of human gut health-promoting conditions (3). However, there are still unsolved issues as to the manufacturing and drug delivery of microorganisms – or products thereof – for effective microbial therapeutics. This Research Topic discusses recent discoveries highlighting how deciphering the host-microbe language may advance such a metabolite-based “postbiotic” approach to which delivery systems are definitely key. Two articles focus on how increasing our understanding of the microbial/host interactions and those articles illustrate future directions in microbial therapeutics. Cai et al. provided insights into the role of the oral microbiota in allergic diseases. As an ecosystem containing a complex microbial ecosystem as well as local organized lymphoid structures, the oral immune cells may modulate systemic immune activation and tolerance. However, the oral host-commensal milieu has not been extensively investigated in allergy. Because colonization by microorganisms early in life significantly affects immune system maturation and allergy development in childhood, they might represent biomarkers that could predict allergy and asthma risk. Moving forward, Du et al. focused on the beneficial effect of administered *Lactobacillus* on common respiratory diseases with a major interest in the mechanism and safety of Lactobacillus in regulating respiratory immunity. Overall, the administration of *Lactobacillus* could be beneficial in improving pulmonary health and its application in treating respiratory diseases deserves more attention. With a view to microbial therapeutics, Wiull et al. exploited a recombinant *Lactiplantibacillus plantarum* delivery vector to improve mucosal vaccination against tuberculosis. Based on previous findings demonstrating the immunogenicity of a recombinant *Lactiplantibacillus plantarum* delivery vector with the hybrid antigen Ag85B-ESAT-6 of tuberculosis anchored to the cell membrane, they went on to show the crucial role of antigen delivery at the most appropriate site in order to improve the vaccine efficacy. Teixeira et al. utilized nanovesicles from *Lactobacillus johnsonii* N6.2 with distinct protein and lipid contents to show a potent immunoregulatory activity in primary human pancreatic islets *via* the activation of the xenobiotic receptor Aryl hydrocarbon Receptor (AhR) and IL-10. This represents a clear example of how deciphering host/microbial crosstalk may result in the development of new therapeutic avenues, the success of which still requires innovative delivery platforms for locally targeting AhR. In the same vein, Wei et al. described a drug delivery platform that can be exploited for new antibacterial therapy. They took advantage of nanomaterials to conceive a nanomaterial-based zinc ion interference therapy to treat infections. Based on the dual characteristics of zinc ions, they may be employed to result in either zinc overloading or deprivation. They showed that this dual characteristic confers unparalleled advantages on zinc ions in terms of antibacterial activity. In addition the nanodelivery system could also serve as a platform for integrating multiple modes of antimicrobial action against pathogens. Lastly, Romero-Pinedo et al. discussed potential therapeutic targets for detrimental inflammatory responses associated with infection and identified signaling lymphocytic activation molecule (SLAM) family 8 as a potentially druggable antimicrobial defense activity target in macrophages to treat infection and inflammation.

Overall, the articles included in this Research Topic highlight important aspects associated with microbial based therapeutics approaches, pointing to areas deserving extended attention and investigation over the next years, as is the case of formulation and targeted delivery of microbes and their products.

## Author contributions

All authors listed have made a substantial, direct, and intellectual contribution to the work and approved it for publication.

## Acknowledgments

We thank all the contributing authors as well as the reviewers for their essential contribution to the realization of this Research Topic.

## Conflict of interest

The authors declare that the research was conducted in the absence of any commercial or financial relationships that could be construed as a potential conflict of interest.

## Publisher’s note

All claims expressed in this article are solely those of the authors and do not necessarily represent those of their affiliated organizations, or those of the publisher, the editors and the reviewers. Any product that may be evaluated in this article, or claim that may be made by its manufacturer, is not guaranteed or endorsed by the publisher.
